# Evidence of Recombinant Strains of Porcine Epidemic Diarrhea Virus, United States, 2013

**DOI:** 10.3201/eid2010.140338

**Published:** 2014-10

**Authors:** Peng-Fei Tian, Yu-Lan Jin, Gang Xing, Ling-Ling Qv, Yao-Wei Huang, Ji-Yong Zhou

**Affiliations:** Key Laboratory of Animal Virology of Ministry of Agriculture, Zhejiang University, Hangzhou, China

**Keywords:** porcine epidemic diarrhea virus, PEDV, pigs, recombination, evolution, viruses, coronavirus, United States, China

## Abstract

To investigate the evolutionary process by which porcine epidemic diarrhea virus (PEDV) in the United States hypothetically descended from strains in China, we analyzed PEDV-positive samples collected in China during January 2012–July 2013. Recombination in 2 strain sublineages was likely associated with identification of PEDV in the United States in 2013.

Porcine epidemic diarrhea virus (PEDV) is an alphacoronavirus that causes enteric disease in swine. The disease, PED, is characterized by acute vomiting and watery diarrhea and causes high mortality rates in newborn piglets (*1*). PED was first reported in 1971 in the United Kingdom and was soon identified in many European and Asian countries (*1*,*2*). Variant PEDV strains that were fatal to young pigs, initially isolated during late 2010 in China and Southeast Asia (*3*,*4*) and in May 2013 in the United States (*5*,*6*), have posed a serious threat to the pork industry. Incidence of PEDV-associated large–scale outbreaks of diarrheal disease in China was reported at 80%–100% in suckling piglets (*3*,*7*) and outbreaks in the United States had spread to 25 states by February 2014 (http://www.aasv.org/news/story.php?id=7038), causing numerous deaths in neonatal piglets (*5*,*6*,*8*,*9*).

How the virus entered the United States remains unknown. A phylogenetic analysis based on available full-length genomic PEDV sequences indicated that all PEDV strains were classified into 2 distinct genogroups: G1 and G2 (*6*). PEDV field strains isolated before 2010 and the derived vaccine strains were in the G1 genogroup, whereas all the new PEDV strains isolated since 2011 in China and the United States (US PEDV) are in G2. The US PEDV sequences were >99% identical to strains found in China in the subgroup G2a, suggesting their origin. In particular, the US PEDV are most closely related to strain AH2012, which was isolated in eastern China and was proposed to have come from multiple recombination events among G2 lineages of PEDV (*6*). Divergence of PEDV is driven by genetic recombination, as in other coronaviruses (*10*). Details of recombination events in the process are needed to investigate origins. To investigate the evolutionary process by which US PEDV strains hypothetically descended from precursors in China, we conducted a molecular epidemiologic analysis using PEDV-positive samples collected from eastern China since 2012.

## The Study

A total of 169 fecal and intestinal samples were collected from pigs with typical PED symptoms on 26 farms in 4 provinces of eastern China during January 2012–July 2013. The rate of PEDV-positive samples was 56.8% (96/169) as had been determined by using reverse transcription PCR (RT-PCR) specific for the spike (S) gene (*11*). From the positive samples, we selected 24 representative samples (Table) to examine. Using RT-PCR, we determined the sequences of the full-length genomic cDNA for the strain CH/ZJCX-1/2012, identified the spike (S) gene for strains CH/ZJQZ-2w/2012 and CH/ZJDX-1/2012, and identified the region encoding structural protein genes by an order of 5′-S-ORF3-E-M-N-3′ (5′-spike protein–open reading frame 3–envelope–membrane–nucleoprotein-3′) for the remaining 21 strains. All primers were designed based on the PEDV MN strain (GenBank accession no. KF468752). We purified and cloned PCR products into a vector using TA cloning. We used Vector NTI software (http://www.lifetechnologies.com/us/en/home/life-science/cloning/vector-nti-software.html) to assemble and analyze the sequences. We performed multiple alignments of S-ORF3-E-M-N, S, ORF3, M, N, and full-length genomes with available sequences from Asia and the United States (*5*,*6*,*8*,*12*) and performed phylogenetic analyses using the MEGA5.2 program (http://www.megasoftware.net/) with the neighbor-joining method.

**Table:** Summary of 24 representative porcine epidemic diarrheal virus sequences in China determined in this study, 2012–2013*

Strain/year	Province of collection	Sequencing region	GenBank accession no.
CH/ZJCX-1/2012	Zhejiang	Full-length	KF840537
CH/ZJHY-2/2012	Zhejiang	S-ORF3-E-M-N	KF840538
CH/ZJJS-Z/2012	Zhejiang	S-ORF3-E-M-N	KF840539
CH/ZJJS-1Z/2012	Zhejiang	S-ORF3-E-M-N	KF840540
CH/JXZS-3H/2012	Jiangxi	S-ORF3-E-M-N	KF840541
CH/JXZS-2H/2012	Jiangxi	S-ORF3-E-M-N	KF840542
CH/ZJXS212/2012	Zhejiang	S-ORF3-E-M-N	KF840543
CH/ZJHZHY-6/2013	Zhejiang	S-ORF3-E-M-N	KF840544
CH/JXJDZ-F/2012	Jiangxi	S-ORF3-E-M-N	KF840545
CH/ZJJS-2Z/2012	Zhejiang	S-ORF3-E-M-N	KF840546
CH/JXZS-3L/2012	Jiangxi	S-ORF3-E-M-N	KF840547
CH/JXZS-1223L/2012	Jiangxi	S-ORF3-E-M-N	KF840548
CH/SDZD-1/2012	Shandong	S-ORF3-E-M-N	KF840549
CH/SDZD-2/2012	Shandong	S-ORF3-E-M-N	KF840850
CH/HuBWHYQ/2012	Hubei	S-ORF3-E-M-N	KF840851
CH/ZJQZ-2/2012	Zhejiang	S-ORF3-E-M-N	KF840852
CH/ZJHZ-1C/2012	Zhejiang	S-ORF3-E-M-N	KF840853
CH/ZJHZ-2C/2012	Zhejiang	S-ORF3-E-M-N	KF840854
CH/JXJDZ-1/2012	Jiangxi	S-ORF3-E-M-N	KF840855
CH/ZJJS-4X/2012	Zhejiang	S-ORF3-E-M-N	KF840856
CH/ZJQZ-2w/2012	Zhejiang	S	KF840857
CH/ZJDX-1/2012	Zhejiang	S	KF840858
CH/JSZL-2/2013	Jiangxi	S-ORF3-E-M-N	KF840861
CH/JSZL-3/2013	Jiangxi	S-ORF3-E-M-N	KF840862
*S, spike protein; ORF, open reading frame; E, envelope: M, membrane; N, nucleoprotein.			

Similar to most of the sequences recently documented in PEDV strains in China and the United States, the S genes of the 24 samples have a 4,161-nt sequence that, compared with the prototype CV777 strain, shows 97.9%–100% sequence identities, and contain 2 notable insertions at amino acids (aa) 56–59 (IGEN) and 139 (N) and a deletion of 2 aa (GK) at aa positions 160 and 161 at the N terminus (*6*). A phylogenetic analysis comparing S genes showed that, based on the complete genome (Technical Appendix Figure 1), all 24 strains were classified into the same group corresponding to G2. However, it is notable that the Chinese sublineage (branch) most closely related to the US PEDV strains did not include the AH2012 strain. Instead, this sublineage contained the strain CH/ZMDZY/11 and 4 other strains determined in this study. Analyses of the phylogenetic trees constructed on the basis of the S-ORF3-E-M-N genes (Figure), ORF3 or M (Technical Appendix Figure 1) also indicated that the AH2012 strain was not closely related to the US branch, relative to the sublineage represented by strains CH/ZMDZY/11, CH/HuBWHYQ/2012, CH/JXZS-1223L/2012, and CH/JXZS-3L/2012 (designated the ZMDZY sublineage hereafter; Figure). The exception is the N gene–based tree, in which the AH2012 was grouped more closely to the sublineage associated with the United States than the strains in the designated ZMDZY sublineage (Technical Appendix Figure 1).

**Figure:**
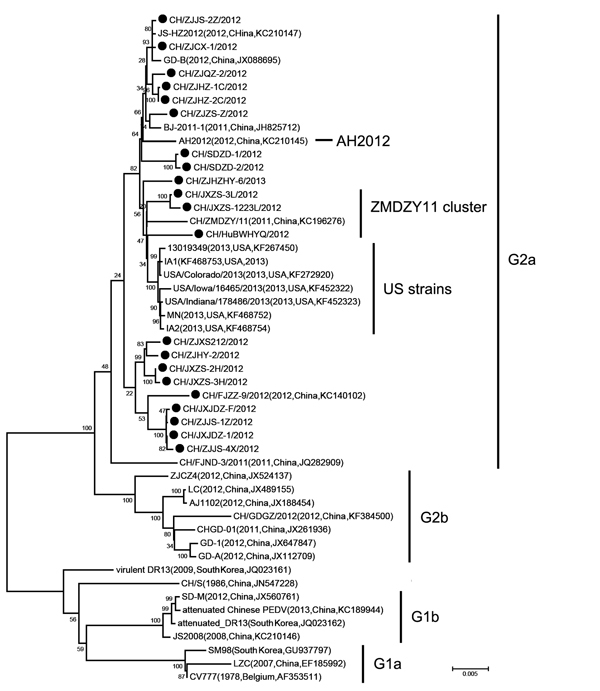
Phylogenetic analysis of newly determined and available porcine epidemic diarrhea virus strains based upon the S-ORF3-E-M-N (5′-spike protein–open reading frame 3–envelope–membrane–nucleoprotein-3′) nucleotide sequences. The tree was constructed by the neighbor-joining method. Bootstrap values are indicated for each node from 1,000 resamplings. The names of the strains, years and places of isolation, GenBank accession numbers, and genogroups proposed by Huang et al. (*6*) are shown. Black solid circles indicate the 21 PEDV strains in this genetic series. Scale bar represents nucleotide substitutions per site.

The relationship of the AH2012 strain with the 33 PEDV strains identified in China, the United States, South Korea, and Belgium (Technical Appendix Figure 1) in nonstructural protein genes was also determined by generation of 3 phylogenetic trees based on ORF1ab, ORF1a, and ORF1b genes, respectively. In accordance with the results from the N gene and the PEDV genotyping based on the full-length genomes (*6*), the AH2012 strain in these trees was most closely related to the US strains (Technical Appendix Figure 1). Therefore, the strains AH2012 and CH/ZMDZY/11 displayed different phylogenetic relationships in different genome regions. Overall, the AH2012 strain was clustered closely with the US strains in the ORF1ab and the N gene region, whereas the ZMDZY sublineage was clustered closely with the US strains in the S-ORF3-E-M region.

To accurately determine how the US strains are related to strains AH2012 and the ZMDZY sublineage, we performed a recombination analysis using the Recombination Detection Program and available PEDV sequences (*13*). We used a multiple-comparison–corrected p-value cutoff of 0.05 in all methods. Recombination events were identified by 6 methods (Recombination Detection Program, GENECONV, BOOTSCAN, MaxChi, CHIMAERA, and SISCAN) when the US PEDV sublineage represented by the MN strain was used as a query. By bootstrap analysis, 3 putatively major recombination breakpoints were detected at nucleotides 6699, 21840, and 26882 (Technical Appendix Figure 2), which generated 2 regions: 1 covered the 3′ half of ORF1a, complete ORF1b, and the N terminus of the S (first 1,207 nt); the other spanned partial S, ORF3, E, M, and partial N (first 504 nt) between the strain AH2012 (as the major parent) and the ZMDZY sublineage (as the minor parent). Although the second region (partial S-ORF3-E-M-partial N) of the US strains is associated with the ZMDZY sublineage, the source of genetic material in this region is not known, because none of the PEDV strains in this sublineage had a highly identical sequence to the consensus sequence of the US strains. It is possible that the other recombination breakpoints exist within the S gene, according to the bootstrap supports in this region, which may be determined by future study with available new sequence data. We showed that the emergent US PEDV strains are possibly descendent of 2 major lineages derived from the ZMDZY sublineage and AH2012 through recombination.

## Conclusion

Our study provides further information on the origin of the US PEDV in 2013. We identified 21 S-ORF3-E-M-N genes, 2 S genes, and 1 full-length genomic cDNA of PEDV from PEDV-positive samples collected in eastern China. Comparative genomic, phylogenetic, and recombination analyses using new and known sequence data demonstrated that the AH2012 strain is likely not the direct progenitor of emergent US PEDV strains during 2013. It is possible that replacement of a region within the partial S-ORF3-E-M-partial N region of the AH2012 strain with a corresponding fragment close to the ZMDZY sublineage (including several newly identified strains) resulted in a recombinant strain related to emergence of this virus in swine in the United States. Other unidentified recombination events and accumulation of adapted mutations within the structural protein genes were also likely involved in this process. 

Technical Appendix Phylogenetic and bootscan analyses of porcine epidemic diarrhea virus isolated in China, South Korea, Japan, and Thailand and in Britain and Belgium in during 1977–2013 to determine origin of strains identified in the United States in 2013.
