# Supplementary material for: Evidence of Recombinant Strains of Porcine Epidemic Diarrhea Virus, United States, 2013
Source: Emerg Infect Dis. 2014 Oct;20(10):1735–8. doi: 10.3201/eid2010.140338 (PMC4193173; doi:10.3201/eid2010.140338)
Supplement: Technical Appendix — Phylogenetic and bootscan analyses of porcine epidemic diarrhea virus isolated in China, South Korea, Japan, and Thailand and in Britain and Belgium in during 1977–2013 to determine origin of strains identified in the United States in 2013. [file 14-0338-Techapp-s1.pdf]

Article DOI: http://dx.doi.org/10.3201/eid2010.140338

Evidence of Recombinant Strains of Porcine Epidemic Diarrhea Virus, United States, 2013

A

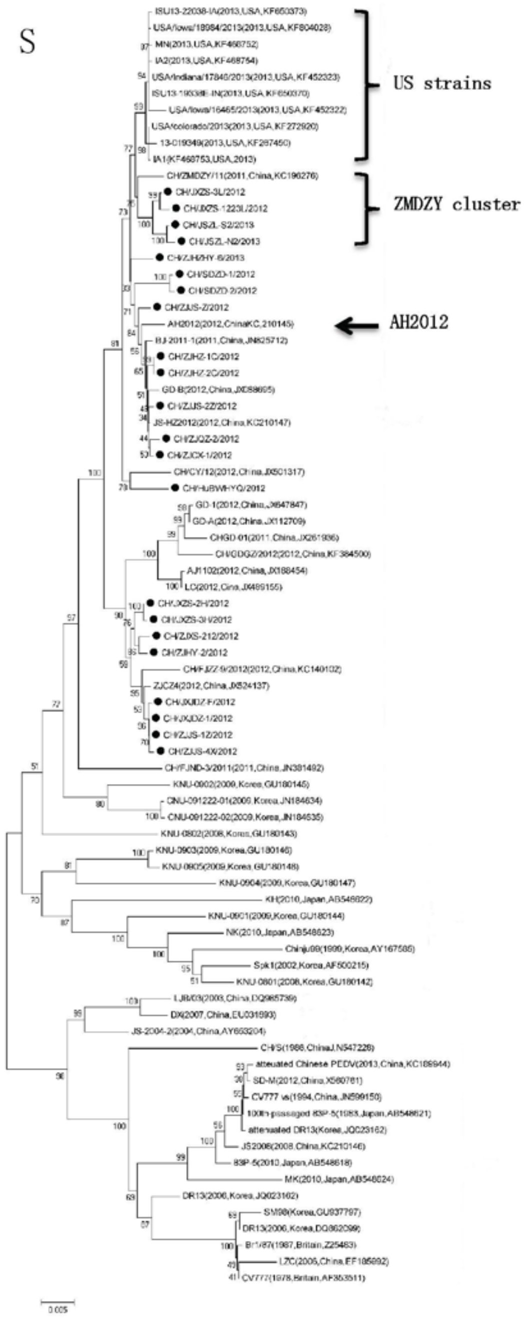

B

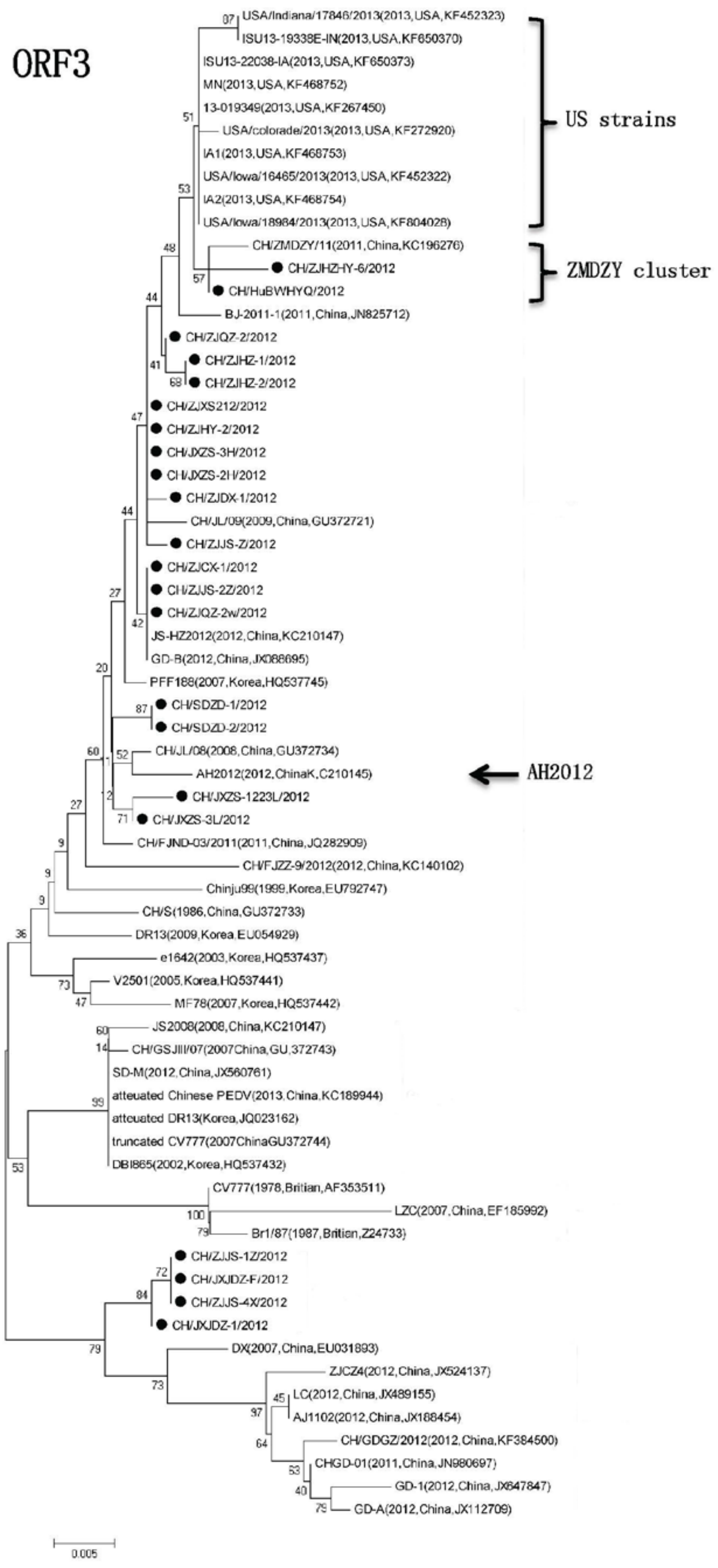
ORF3
USA/Indiana/17846/2013(2013,USA,KF452323)
ISU13-19338E-IN(2013,USA,KF650370)
ISU13-22038-IA(2013,USA,KF650373)
MN(2013,USA,KF468752)
13-019349(2013,USA,KF267450)
USA/colorade/2013(2013,USA,KF272920)
IA1(2013,USA,KF468753)
USA/Iowa/16465/2013(2013,USA,KF452322)
IA2(2013,USA,KF468754)
USA/Iowa/18984/2013(2013,USA,KF804028)
US strains
CH/ZMDZY/11(2011,China,KC196276)
CH/ZJHZHY-6/2012
CH/HuBWHYQ/2012
ZMDZY cluster
BJ-2011-1(2011,China,JN825712)
CH/ZJQZ-2/2012
CH/ZJHZ-1/2012
CH/ZJHZ-2/2012
CH/ZJXS212/2012
CH/ZJHY-2/2012
CH/JXZS-3H/2012
CH/JXZS-2H/2012
CH/ZJDX-1/2012
CH/JL/09(2009,China,GU372721)
CH/ZJJS-Z/2012
CH/ZJCX-1/2012
CH/ZJJS-2Z/2012
CH/ZJQZ-2w/2012
JS-HZ2012(2012,China,KC210147)
GD-B(2012,China,JX088695)
PFF188(2007,Korea,HQ537745)
CH/SDZD-1/2012
CH/SDZD-2/2012
CH/JL/08(2008,China,GU372734)
AH2012(2012,ChinaK,C210145)
AH2012
CH/JXZS-1223L/2012
CH/JXZS-3L/2012
CH/FJND-03/2011(2011,China,JQ282909)
CH/FJZZ-9/2012(2012,China,KC140102)
Chinju99(1999,Korea,EU792747)
CH/S(1986,China,GU372733)
DR13(2009,Korea,EU054929)
e1642(2003,Korea,HQ537437)
V2501(2005,Korea,HQ537441)
MF78(2007,Korea,HQ537442)
JS2008(2008,China,KC210147)
CH/GSJIII/07(2007China,GU,372743)
SD-M(2012,China,JX560761)
atteuated Chinese PEDV(2013,China,KC189944)
atteuated DR13(Korea,JQ023162)
truncated CV777(2007ChinaGU372744)
DBI865(2002,Korea,HQ537432)
CV777(1978,Britian,AF353511)
LZC(2007,China,EF185992)
Br1/87(1987,Britian,Z24733)
CH/ZJJS-1Z/2012
CH/JXJDZ-F/2012
CH/ZJJS-4X/2012
CH/JXJDZ-1/2012
DX(2007,China,EU031893)
ZJCZ4(2012,China,JX524137)
LC(2012,China,JX489155)
AJ1102(2012,China,JX188454)
CH/GDGZ/2012(2012,China,KF384500)
CHGD-01(2011,China,JN980697)
GD-1(2012,China,JX647847)
GD-A(2012,China,JX112709)
0.005

C

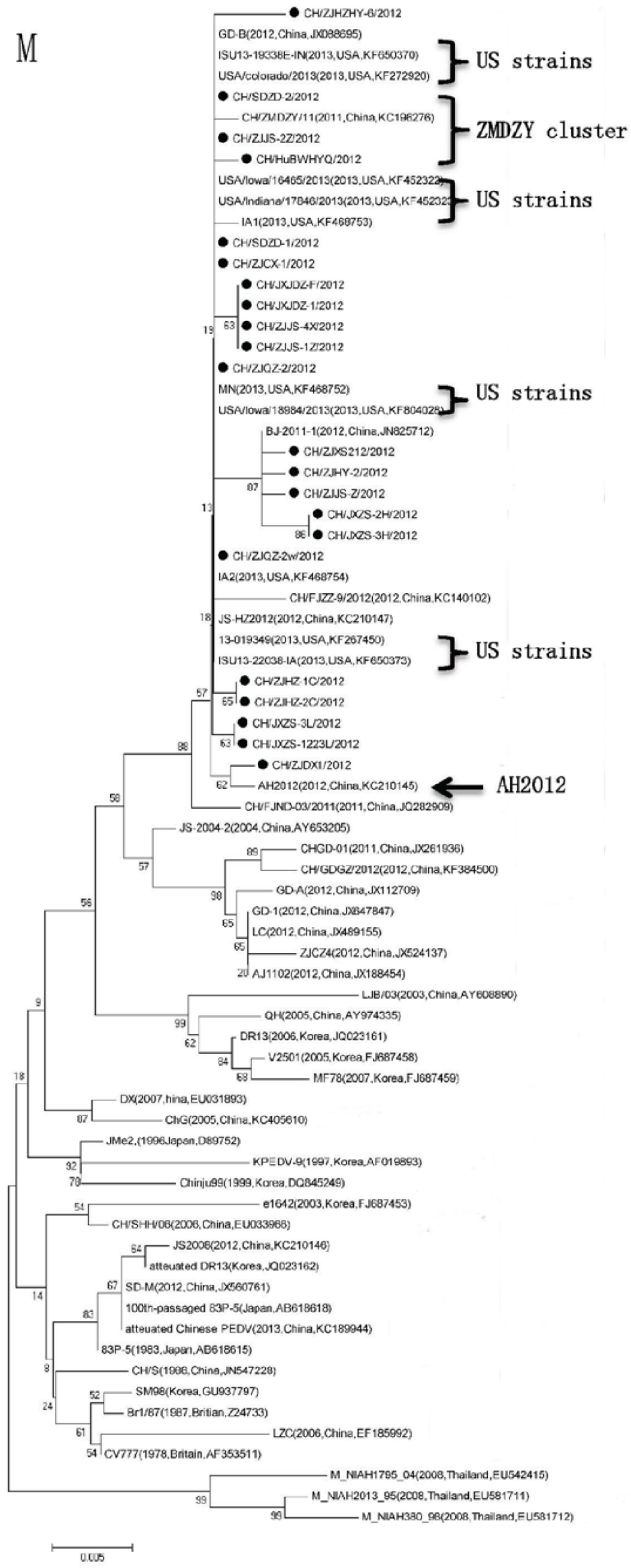
M
US strains
ZMDZY cluster
US strains
US strains
US strains
AH2012
CH/ZJHZHY-6/2012
GD-B(2012,China,JX088695)
ISU13-19338E-IN(2013,USA,KF650370)
USA/colorado/2013(2013,USA,KF272920)
CH/SDZD-2/2012
CH/ZMDZY/11(2011,China,KC196276)
CH/ZJJS-2Z/2012
CH/HuBWHYQ/2012
CH/SDZD-1/2012
CH/ZJCX-1/2012
CH/JXJDZ-F/2012
CH/JXJDZ-1/2012
CH/ZJJS-4X/2012
CH/ZJJS-1Z/2012
CH/ZJQZ-2/2012
MN(2013,USA,KF468752)
BJ-2011-1(2012,China,JN825712)
CH/ZJXS212/2012
CH/ZJHY-2/2012
CH/ZJJS-Z/2012
CH/JXZS-2H/2012
CH/JXZS-3H/2012
CH/ZJQZ-2w/2012
IA2(2013,USA,KF468754)
CH/FJZZ-9/2012(2012,China,KC140102)
JS-HZ2012(2012,China,KC210147)
13-019349(2013,USA,KF267450)
ISU13-22038-IA(2013,USA,KF650373)
CH/ZJHZ-1C/2012
CH/ZJHZ-2C/2012
CH/JXZS-3L/2012
CH/JXZS-1223L/2012
CH/ZJDX1/2012
AH2012(2012,China,KC210145)
CH/FJND-03/2011(2011,China,JQ282909)
JS-2004-2(2004,China,AY653205)
CHGD-01(2011,China,JX261936)
CH/GDGZ/2012(2012,China,KF384500)
GD-A(2012,China,JX112709)
GD-1(2012,China,JX647847)
LC(2012,China,JX489155)
ZJCZ4(2012,China,JX524137)
AJ1102(2012,China,JX188454)
LJB/03(2003,China,AY608890)
QH(2005,China,AY974335)
DR13(2006,Korea,JQ023161)
V2501(2005,Korea,FJ687458)
MF78(2007,Korea,FJ687459)
DX(2007,hina,EU031893)
ChG(2005,China,KC405610)
JMe2,(1996Japan,D89752)
KPEDV-9(1997,Korea,AF019893)
Chinju99(1999,Korea,DQ845249)
e1642(2003,Korea,FJ687453)
CH/SHH/06(2006,China,EU033966)
JS2008(2012,China,KC210146)
atteuated DR13(Korea,JQ023162)
SD-M(2012,China,JX560761)
100th-passaged 83P-5(Japan,AB618618)
atteuated Chinese PEDV(2013,China,KC189944)
83P-5(1983,Japan,AB618615)
CH/S(1986,China,JN547228)
SM98(Korea,GU937797)
Br1/87(1987,Britian,Z24733)
LZC(2006,China,EF185992)
CV777(1978,Britain,AF353511)
M_NIAH1795_04(2008,Thailand,EU542415)
M_NIAH2013_95(2008,Thailand,EU581711)
M_NIAH380_98(2008,Thailand,EU581712)
0.005

D

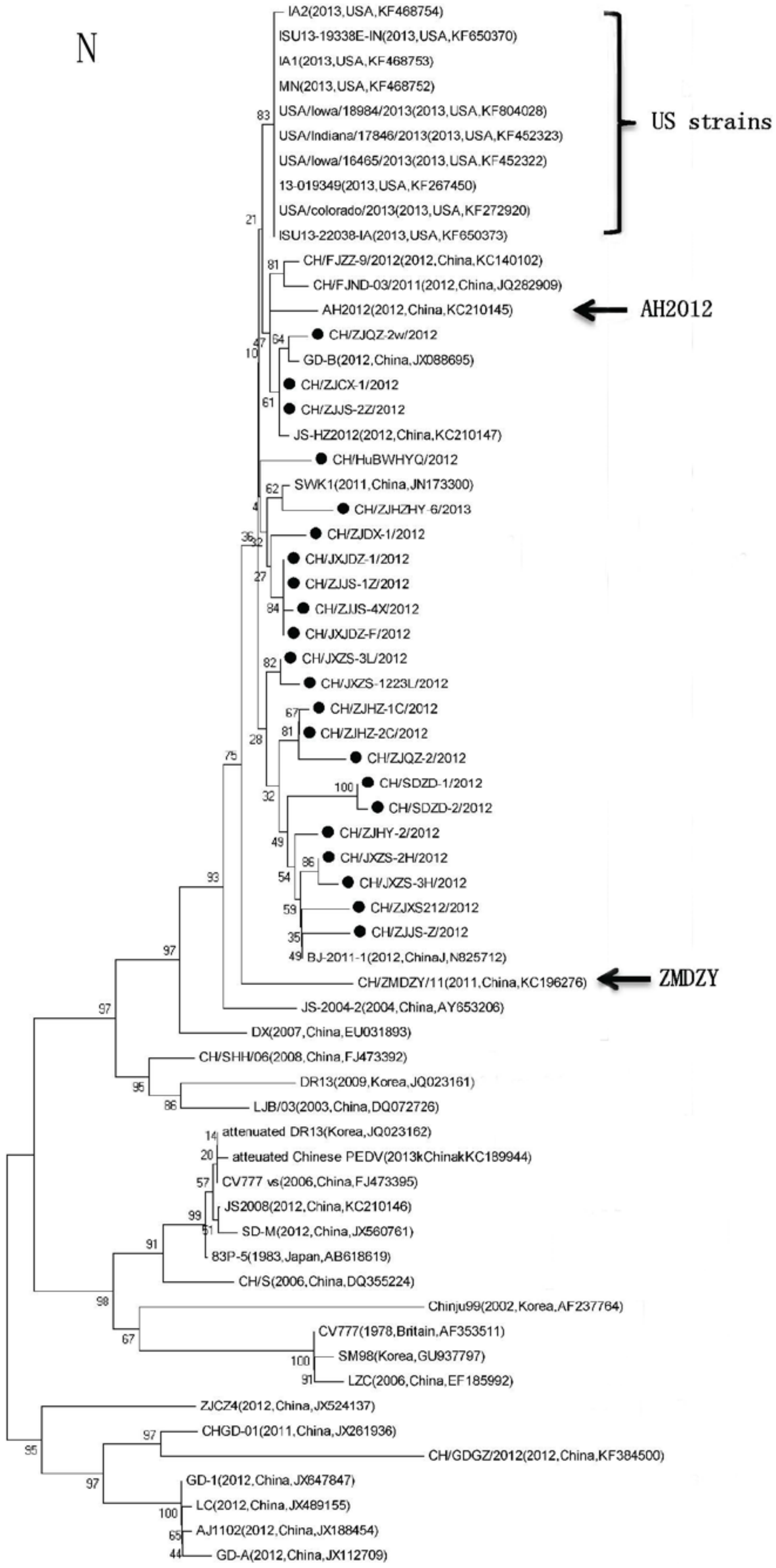
N
IA2(2013,USA,KF468754)
ISU13-19338E-IN(2013,USA,KF650370)
IA1(2013,USA,KF468753)
MN(2013,USA,KF468752)
USA/Iowa/18984/2013(2013,USA,KF804028)
USA/Indiana/17846/2013(2013,USA,KF452323)
USA/Iowa/16465/2013(2013,USA,KF452322)
13-019349(2013,USA,KF267450)
USA/colorado/2013(2013,USA,KF272920)
ISU13-22038-IA(2013,USA,KF650373)
US strains
CH/FJZZ-9/2012(2012,China,KC140102)
CH/FJND-03/2011(2012,China,JQ282909)
AH2012(2012,China,KC210145)
AH2012
CH/ZJQZ-2w/2012
GD-B(2012,China,JX088695)
CH/ZJCX-1/2012
CH/ZJJS-2Z/2012
JS-HZ2012(2012,China,KC210147)
CH/HuBWHYQ/2012
SWK1(2011,China,JN173300)
CH/ZJHZHY-6/2013
CH/ZJDX-1/2012
CH/JXJDZ-1/2012
CH/ZJJS-1Z/2012
CH/ZJJS-4X/2012
CH/JXJDZ-F/2012
CH/JXZS-3L/2012
CH/JXZS-1223L/2012
CH/ZJHZ-1C/2012
CH/ZJHZ-2C/2012
CH/ZJQZ-2/2012
CH/SDZD-1/2012
CH/SDZD-2/2012
CH/ZJHY-2/2012
CH/JXZS-2H/2012
CH/JXZS-3H/2012
CH/ZJXS212/2012
CH/ZJJS-Z/2012
BJ-2011-1(2012,ChinaJ,N825712)
CH/ZMDZY/11(2011,China,KC196276)
ZMDZY
JS-2004-2(2004,China,AY653206)
DX(2007,China,EU031893)
CH/SHH/06(2008,China,FJ473392)
DR13(2009,Korea,JQ023161)
LJB/03(2003,China,DQ072726)
attenuated DR13(Korea,JQ023162)
atteuated Chinese PEDV(2013kChinakKC189944)
CV777 vs(2006,China,FJ473395)
JS2008(2012,China,KC210146)
SD-M(2012,China,JX560761)
83P-5(1983,Japan,AB618619)
CH/S(2006,China,DQ355224)
Chinju99(2002,Korea,AF237764)
CV777(1978,Britain,AF353511)
SM98(Korea,GU937797)
LZC(2006,China,EF185992)
ZJCZ4(2012,China,JX524137)
CHGD-01(2011,China,JX261936)
CH/GDGZ/2012(2012,China,KF384500)
GD-1(2012,China,JX647847)
LC(2012,China,JX489155)
AJ1102(2012,China,JX188454)
GD-A(2012,China,JX112709)

E

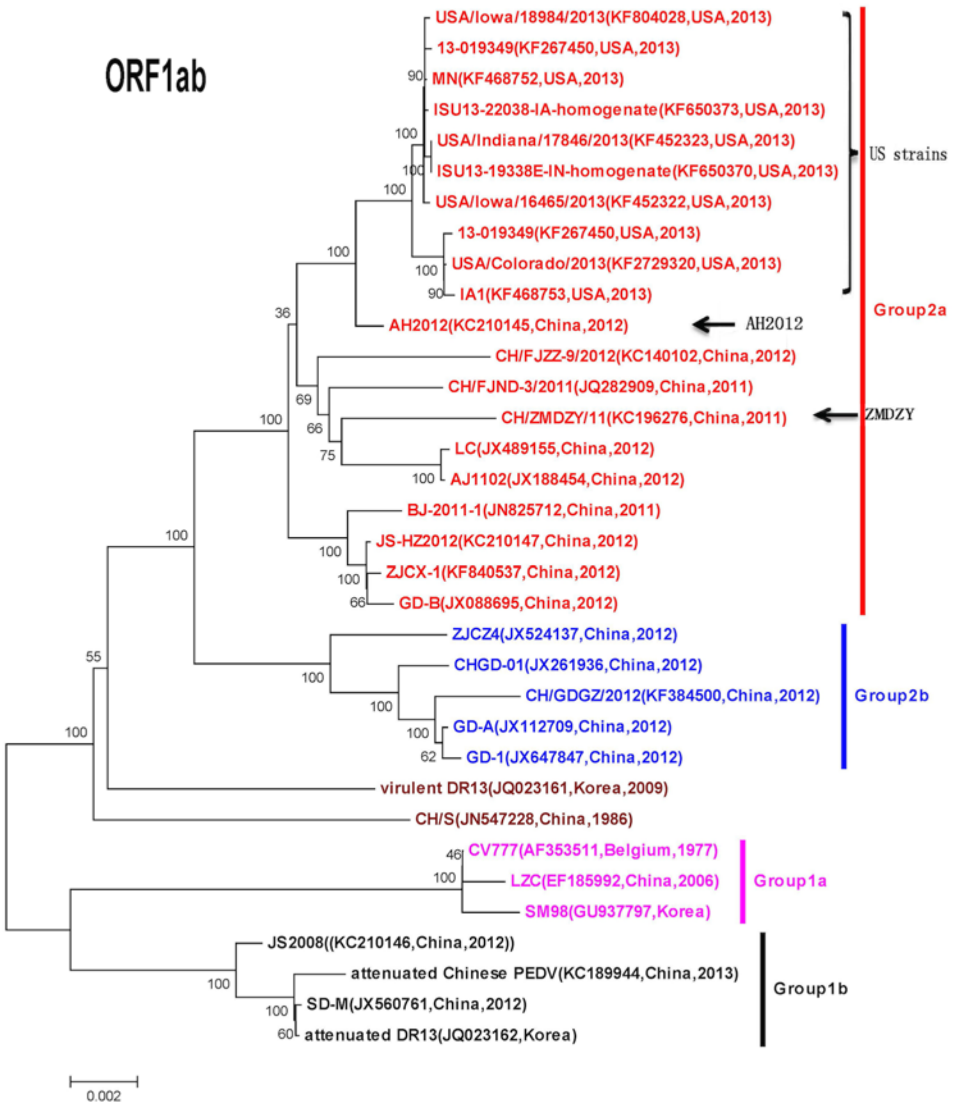
ORF1ab
USA/Iowa/18984/2013(KF804028,USA,2013)
13-019349(KF267450,USA,2013)
MN(KF468752,USA,2013)
ISU13-22038-IA-homogenate(KF650373,USA,2013)
USA/Indiana/17846/2013(KF452323,USA,2013)
ISU13-19338E-IN-homogenate(KF650370,USA,2013)
USA/Iowa/16465/2013(KF452322,USA,2013)
13-019349(KF267450,USA,2013)
USA/Colorado/2013(KF2729320,USA,2013)
IA1(KF468753,USA,2013)
AH2012(KC210145,China,2012)
CH/FJZZ-9/2012(KC140102,China,2012)
CH/FJND-3/2011(JQ282909,China,2011)
CH/ZMDZY/11(KC196276,China,2011)
LC(JX489155,China,2012)
AJ1102(JX188454,China,2012)
BJ-2011-1(JN825712,China,2011)
JS-HZ2012(KC210147,China,2012)
ZJCX-1(KF840537,China,2012)
GD-B(JX088695,China,2012)
ZJCZ4(JX524137,China,2012)
CHGD-01(JX261936,China,2012)
CH/GDGZ/2012(KF384500,China,2012)
GD-A(JX112709,China,2012)
GD-1(JX647847,China,2012)
virulent DR13(JQ023161,Korea,2009)
CH/S(JN547228,China,1986)
CV777(AF353511,Belgium,1977)
LZC(EF185992,China,2006)
SM98(GU937797,Korea)
JS2008((KC210146,China,2012))
attenuated Chinese PEDV(KC189944,China,2013)
SD-M(JX560761,China,2012)
attenuated DR13(JQ023162,Korea)
US strains
AH2012
ZMDZY
Group2a
Group2b
Group1a
Group1b
0.002

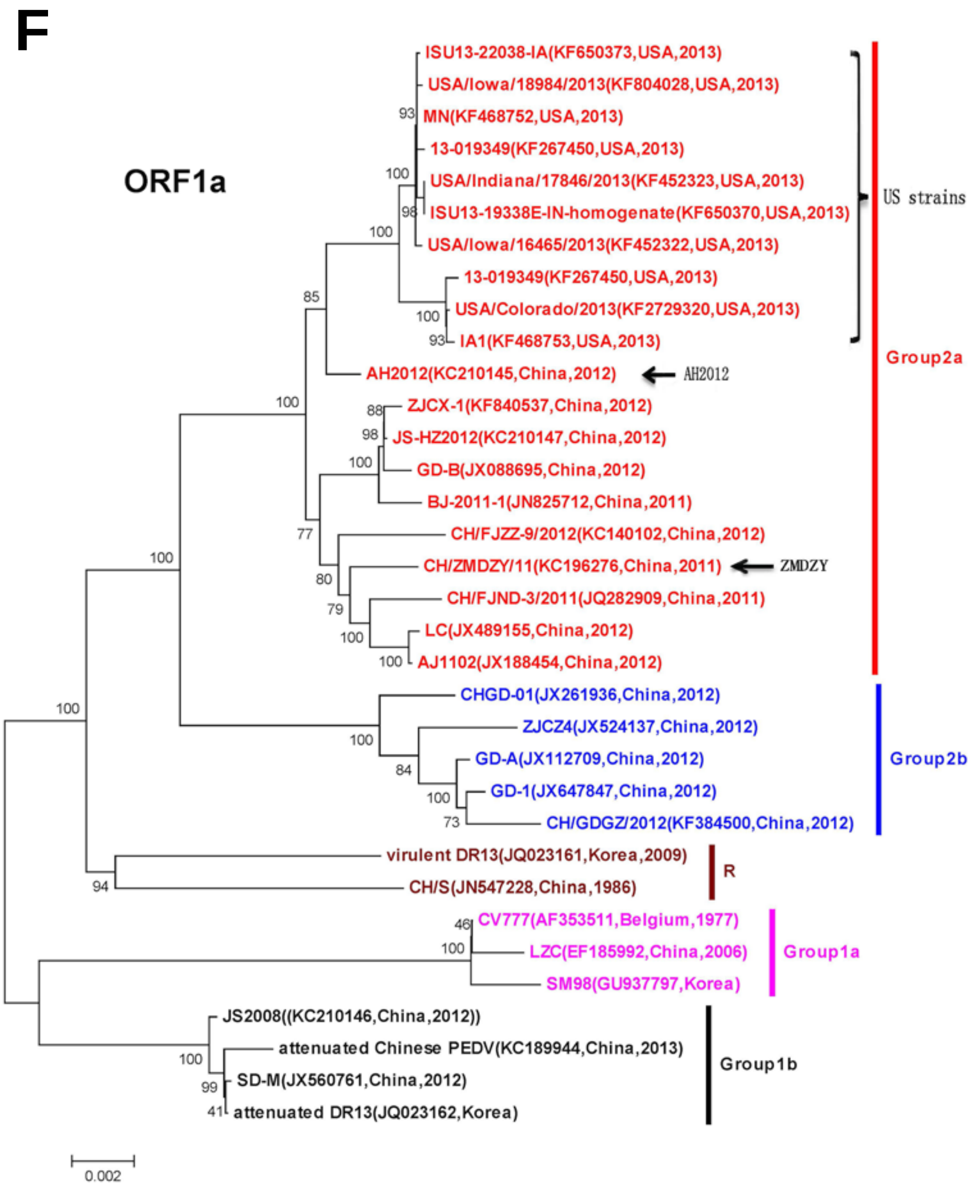
F
ORF1a
ISU13-22038-IA(KF650373,USA,2013)
USA/Iowa/18984/2013(KF804028,USA,2013)
MN(KF468752,USA,2013)
13-019349(KF267450,USA,2013)
USA/Indiana/17846/2013(KF452323,USA,2013)
ISU13-19338E-IN-homogenate(KF650370,USA,2013)
USA/Iowa/16465/2013(KF452322,USA,2013)
13-019349(KF267450,USA,2013)
USA/Colorado/2013(KF2729320,USA,2013)
IA1(KF468753,USA,2013)
AH2012(KC210145,China,2012)
AH2012
ZJCX-1(KF840537,China,2012)
JS-HZ2012(KC210147,China,2012)
GD-B(JX088695,China,2012)
BJ-2011-1(JN825712,China,2011)
CH/FJZZ-9/2012(KC140102,China,2012)
CH/ZMDZY/11(KC196276,China,2011)
ZMDZY
CH/FJND-3/2011(JQ282909,China,2011)
LC(JX489155,China,2012)
AJ1102(JX188454,China,2012)
US strains
Group2a
CHGD-01(JX261936,China,2012)
ZJCZ4(JX524137,China,2012)
GD-A(JX112709,China,2012)
GD-1(JX647847,China,2012)
CH/GDGZ/2012(KF384500,China,2012)
Group2b
virulent DR13(JQ023161,Korea,2009)
CH/S(JN547228,China,1986)
R
CV777(AF353511,Belgium,1977)
LZC(EF185992,China,2006)
SM98(GU937797,Korea)
Group1a
JS2008((KC210146,China,2012))
attenuated Chinese PEDV(KC189944,China,2013)
SD-M(JX560761,China,2012)
attenuated DR13(JQ023162,Korea)
Group1b
0.002

G

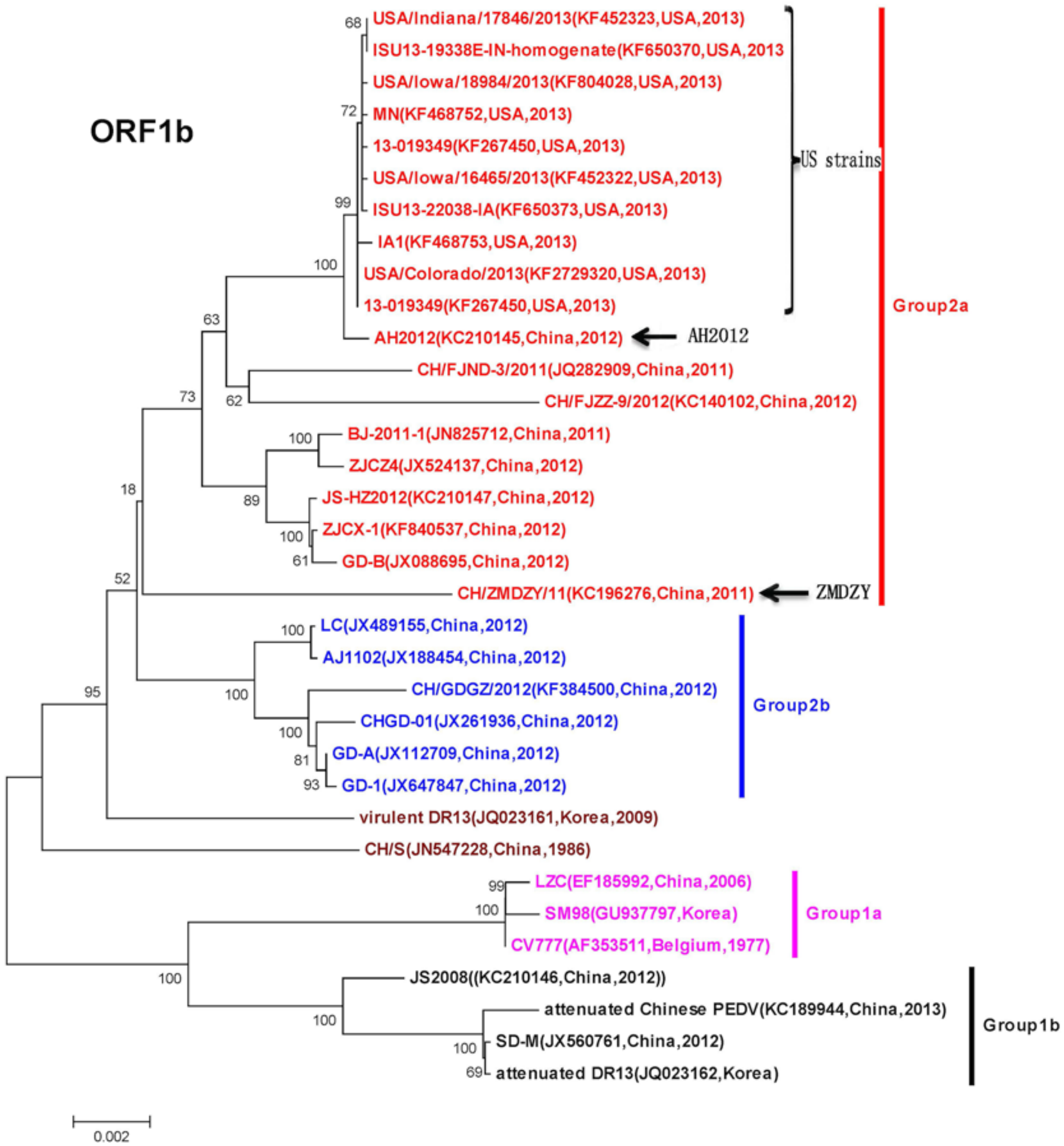
ORF1b
USA/Indiana/17846/2013(KF452323,USA,2013)
ISU13-19338E-IN-homogenate(KF650370,USA,2013
USA/Iowa/18984/2013(KF804028,USA,2013)
MN(KF468752,USA,2013)
13-019349(KF267450,USA,2013)
USA/Iowa/16465/2013(KF452322,USA,2013)
ISU13-22038-IA(KF650373,USA,2013)
IA1(KF468753,USA,2013)
USA/Colorado/2013(KF2729320,USA,2013)
13-019349(KF267450,USA,2013)
US strains
AH2012(KC210145,China,2012)
AH2012
CH/FJND-3/2011(JQ282909,China,2011)
CH/FJZZ-9/2012(KC140102,China,2012)
BJ-2011-1(JN825712,China,2011)
ZJCZ4(JX524137,China,2012)
JS-HZ2012(KC210147,China,2012)
ZJCX-1(KF840537,China,2012)
GD-B(JX088695,China,2012)
CH/ZMDZY/11(KC196276,China,2011)
ZMDZY
Group2a
LC(JX489155,China,2012)
AJ1102(JX188454,China,2012)
CH/GDGZ/2012(KF384500,China,2012)
CHGD-01(JX261936,China,2012)
GD-A(JX112709,China,2012)
GD-1(JX647847,China,2012)
Group2b
virulent DR13(JQ023161,Korea,2009)
CH/S(JN547228,China,1986)
LZC(EF185992,China,2006)
SM98(GU937797,Korea)
CV777(AF353511,Belgium,1977)
Group1a
JS2008((KC210146,China,2012))
attenuated Chinese PEDV(KC189944,China,2013)
SD-M(JX560761,China,2012)
attenuated DR13(JQ023162,Korea)
Group1b
0.002

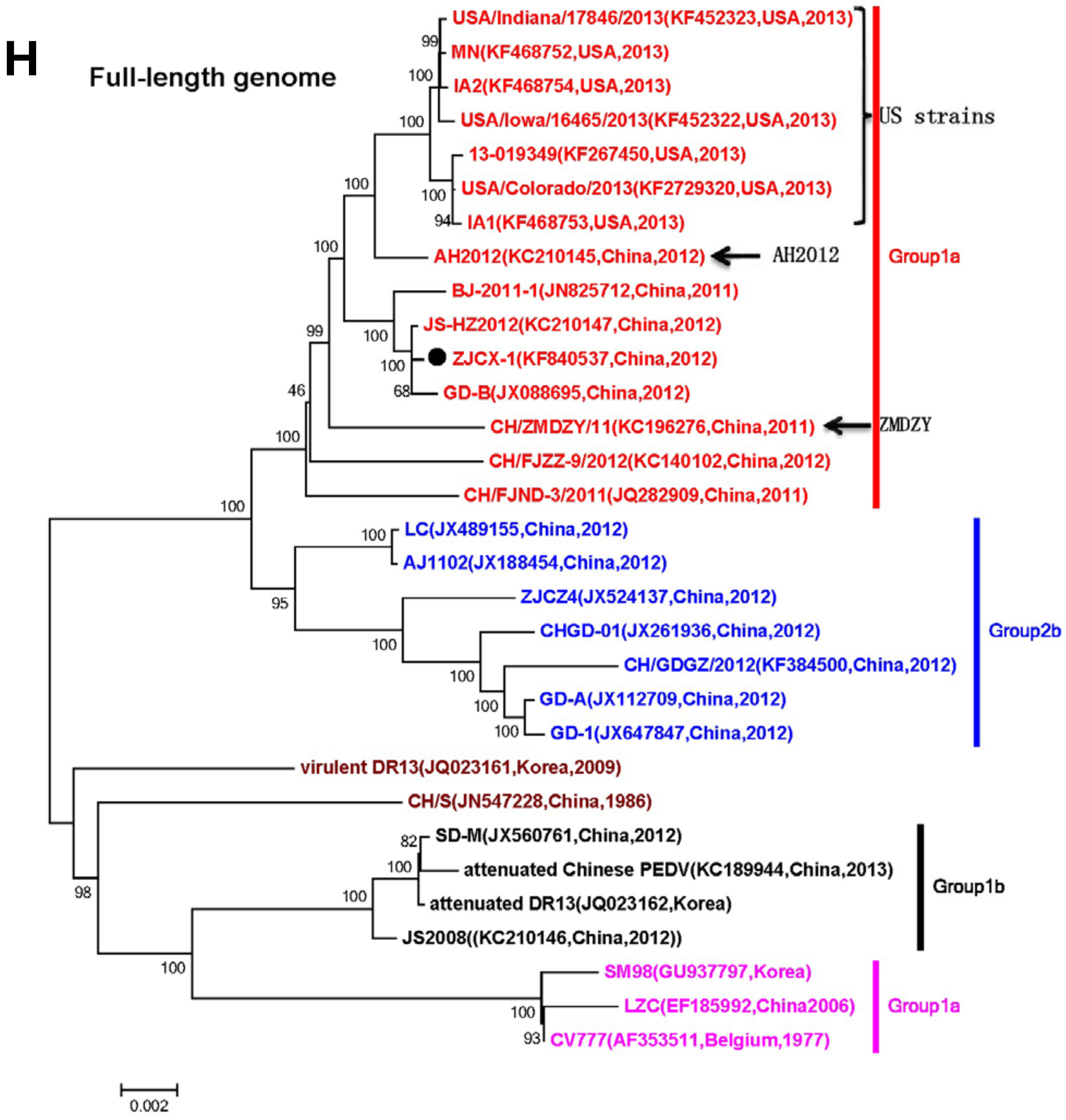

Technical Appendix Figure 1. Phylogenetic analyses of newly determined and available porcine epidemic diarrhea virus (PEDV) strains based upon nucleotide sequences of genes as follows: A) S; B) ORF3; C) M; D) N; E) ORF1ab; F) ORF1a; G) ORF1b genes; and H) the full-length genome. The trees were constructed by the neighbor-joining method. Bootstrap values are indicated for each node from 1,000 resamplings. The names of the strains, years and places of isolation, and GenBank accession numbers are shown. Black solid circles indicate the new PEDV strains sequenced in this study. Scale bars represent nucleotide substitutions per site.

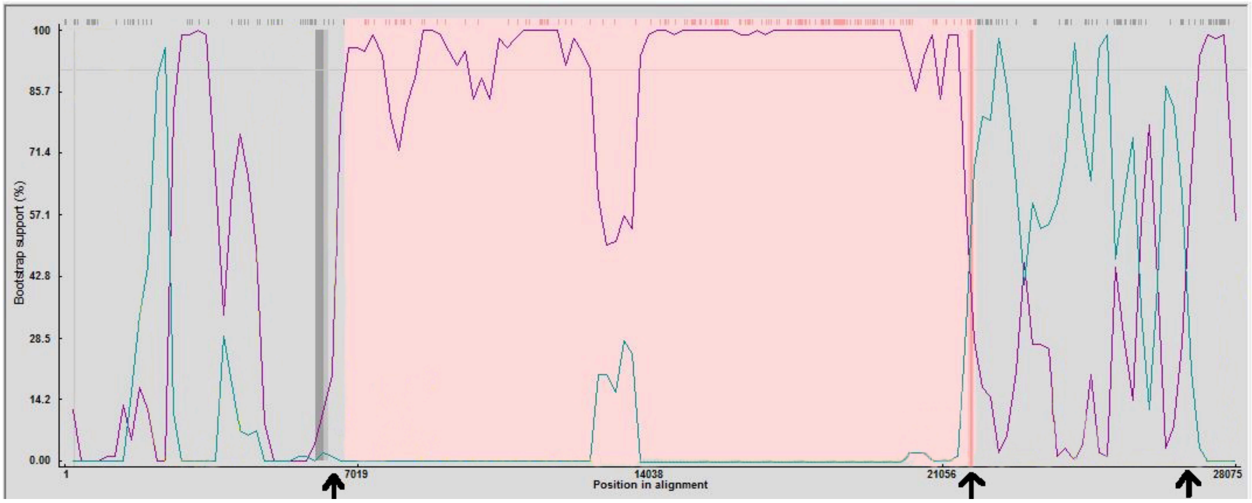

Technical Appendix Figure 2. Detection of potential recombination events in the PEDV strains identified in the United States by bootstrap analysis. Three major recombination breakpoints, denoted by arrows, were located, respectively, at the ORF1a (nt 6,699), S (nt 21,840) and N (nt 26,882) genes (based on the MN strain) (*6*). The analysis was performed with an F84 distance model, a window size of 1,000 base pairs and a step size of 200 base pairs. Regions derived from the AH2012 or the ZMDZY sublineage is denoted by purple or cyan lines, respectively.
